# Attenuation of self-produced touch in the posterior insula reflects body image disturbances in anorexia nervosa

**DOI:** 10.1038/s41598-026-71154-5

**Published:** 2026-09-26

**Authors:** Morgan Frost-Karlsson, Maria Zetterqvist, Håkan Olausson, Rebecca Boehme

**Affiliations:** 1 Department of Biomedical and Clinical Sciences, Center for Social and Affective Neuroscience, Linköping University, S-581 83 Linköping, Sweden; 2 Clinical Department of Child and Adolescent Psychiatry in Linköping, Region Östergötland, Linköping, Sweden; 3 Department of Clinical Neurophysiology, Linköping University Hospital, 58185 Linköping, Sweden; 4 Center for Medical Imaging and Visualization, Linköping University, 58185 Linköping, Sweden

**Keywords:** Prediction errors, Bodily self, fMRI, Anorexia nervosa, Body image disturbance, Insula, Neuroscience, Psychology, Psychology

## Abstract

Body image disturbance (BID) is a core feature of anorexia nervosa (AN), encompassing multimodal distortions in bodily self-perception. Affective touch, a gentle, emotionally salient tactile stimulus, is implicated in body ownership but has not been extensively studied in AN. Affective touch is processed in the insula, an area involved in interoception and bodily self-awareness which has been shown to be altered both structurally and functionally in AN. This study explored the neural mechanisms underlying BID by examining how individuals with AN process self-produced affective touch. Women with AN (*n* = 44) and age-matched healthy controls (HC, *n* = 40), participated in a self-other-object touch task during functional brain imaging and filled out questionnaires related to body image and eating disorder symptomatology. During self-touch, individuals with AN exhibited increased activation in the right posterior insula compared to object-touch, whereas HC did not. This insular activation correlated positively with body attitude (BAT) and body size estimation (FRS) scores in AN, but negatively in healthy controls, suggesting divergent processing of self-related tactile input. These findings suggest that posterior insular responses to self-produced touch may reflect disturbed sensory self-processing in AN, and that touch-based mechanisms contributing to body ownership in healthy individuals may operate differently in the context of AN. Broadening BID research beyond the visual modality opens new avenues for translational research and treatment.

**Supplementary Information:**

The online version contains supplementary material available at https://doi.org/10.1038/s41598-026-71154-5.

## Introduction

Body image disturbance (BID) is one of the hallmark features of anorexia nervosa (AN), a severe eating disorder characterized by distorted body perception, weight loss, and intense fear of gaining weight. Individuals with AN consistently perceive their bodies as larger than they are and engage in extreme behaviors to control their weight. Individuals with AN perceive their bodies as larger than reality but this is not merely a visual misestimation, they *feel* their bodies as larger across modalities (individuals with AN overestimate tactile distances on their bodies^1^, and can even turn their shoulders to fit through a doorway^2^, . These misperceptions are specific to the self, not bodies in general, and are intricately linked to neural mechanisms of bodily self-awareness, interoception, and affect regulation^3^. Despite this multimodal character, most research focuses on the visual modality and thus fails to encapsulate the full nature of BID^4^.

Affective touch, characterized by gentle, caress-like tactile stimulation, plays a crucial role in the development and maintenance of our sense of self and body ownership. Affective touch is primarily mediated by C-tactile (CT) afferents, specialized nerve fibers that respond optimally to slow, gentle stroking^5^. Research on affective touch in AN is limited but suggests that women with AN experience a tactile hypersensitivity which correlates with body image disturbance^6^. CT-mediated touch has also been found to be less pleasant in AN compared to healthy women, which persists even after recovery^7,8^. Notably, a recent study found differential modulation of multisensory body processing in AN and highlights the need for research and treatment incorporating components of bodily self-awareness^4^.

While affective touch delivered by others has received some attention in AN^9^, self-produced affective touch is theoretically distinct, because it involves both the efference copy of the motor command and the resulting somatosensory input, making it more directly related to the brain’s predictive self-model^10^. Discrepancies between predicted and actual self-touch signals would be expected to reflect inaccuracies in the bodily self-model, and thus affective self-touch may be a particularly sensitive measurement for BID, engaging the mechanisms thought to underlie body ownership and embodiment^11^. Recent studies examining affective and social touch in AN have explored how interpersonal and self-directed touch experiences diverge in this population^12,13^.

CT-mediated tactile stimulation activates the contralateral posterior insular cortex^5,14^. The insula integrates sensory information from the body with emotional processing, helping form a cohesive sense of self^15^. Functional imaging studies have consistently shown altered insular structure and activity in individuals with AN (see, e.g.^16–22^, with some structural alterations persisting after recovery^16^. The posterior insula is associated with sensorimotor integration, interoception^23^, changes in interoceptive states and somatosensory processes^24^. Given the posterior insula’s role in interoception and body ownership, altered posterior insular structure and/or function could potentially be an important marker for pathophysiology in AN both before onset and after recovery^18^.

Our previous research in healthy populations has found attenuation of self-produced touch in the insula (among other areas)^25^, while AN had greater activation across multiple brain regions during self-touch compared to both controls and adults on the Autism Spectrum^9^, a pattern we interpret as reflecting prediction errors arising from an inaccurate bodily self-model.

Under the Predictive Coding Framework, the brain continuously generates predictions about sensory input and updates its internal model when prediction errors arise^26^. This process might be altered in AN. According to the Allocentric Lock Hypothesis^27^, individuals with AN have difficulties integrating multisensory information and struggle to perceive their bodies accurately from their own perspective, which leads to them being “locked” in a distorted body schema which does not update according to accurate sensory input. From a predictive coding perspective, this manifests as persistent prediction errors, as sensory input about one’s own body is continuously mismatched with a flawed self-model. Consistent with this, neuroimaging studies have shown increased prediction errors in AN in regard to taste reward^28^, as well as increased sensitivity to salient stimuli and greater intolerance of uncertainty^29^.

BID precedes onset of AN^30^ and may contribute to poor outcome and relapse^31^. As BID is such a dominant and predictive feature of AN, studying its mechanisms is necessary for deeper understanding of AN. Legrand^11^ argues that BID in AN is not merely an overestimation of size but a specifically self-referential distortion: the body representation is oversized relative to one’s own body, but not relative to others’ bodies, implicating self-attribution and self-representation as central mechanisms that remain underexplored. Understanding BID thus requires paradigms that engage self-specific bodily processing. Body image components in eating disorders are multidimensional, spanning perceptual, affective, and cognitive dimensions^32^, and studying their neural correlates requires measures that capture this breadth. Relapse is prevalent in AN^33^ and studying the mechanisms of BID can inform new treatment options.

In this study we measured insular activation using functional magnetic resonance imaging (fMRI) during self-produced affective touch (slow stroking). Stroking was chosen as the stimulus modality because it optimally activates CT afferents (affective touch processing), while the self-produced nature of the touch (predictable) engages predictive processing. We assessed this activation in relation to measures of BID. We hypothesized that AN would show greater insular activation than healthy controls (HC) during self-touch. Additionally, we included measures during other-touch to allow characterization of interpersonal, passive affective touch responses and is reported as a secondary, exploratory analysis. By focusing on this key but understudied modality, we aimed to extend body image research beyond vision and identify potential neural markers of disturbed embodiment in AN.

## Results

### Participant demographics & questionnaires

In total, 44 women with AN and 39 HC were analyzed. Groups were matched in age (AN: M = 21.4 years, SD = 3.4; HC: M = 21.9 years, SD = 2.8) but differed significantly in BMI (AN: M = 19.0, SD = 1.3; HC: M = 21.8, SD = 2.4, *p* < 0.001). As expected, the AN group reported markedly higher body image disturbance and eating disorder symptomatology than HC. On BAT, AN scored well above the clinical cut-off of 36^34^, (M = 63.6, SD = 16.2) compared to HC (M = 19.1, SD = 9.6, *p* < 0.001). A similar pattern emerged on EDE-Q, in which cut-off is 2.8^35^(AN: M = 13.7, SD = 19.9; HC: M = 0.9, SD = 1.5, *p* < 0.001), and on FRS (AN: M = 2.07, SD = 1.96; HC: M = 0.46, SD = 0.8, *p* < 0.001).

For demographic and questionnaire values, see Table 1.

**Table 1:** Participant demographics and questionnaire results (means and standard deviations).

	AN (*N* = 44)	HC (*N* = 39)	t/W-value; *p*-value
Age	21.425 (3.4)	21.9 (2.8)	t = 0.689; *p* = 0.493
BMI	19 (1.3)	21.8 (2.4)	t = 6.711; *p* < 0.001
BAT	63.58 (16.24)	19.11(9.6)	W = 19.5; *p* < 0.001
FRS	2.07 (1.96)	0.46(0.8)	W = 338.5; *p* < 0.001
EDE-Q	13.65 (19.9)	0.88(1.5)	W = 29; *p* < 0.001

*AN* anorexia nervosa, *HC* healthy control, *BMI* body mass index, *BAT* body attitude test, *FRS* figure rating scale, *EDE-Q* eating disorders examination questionnaire.

### Main effects of touch condition

**Fig. 1:**
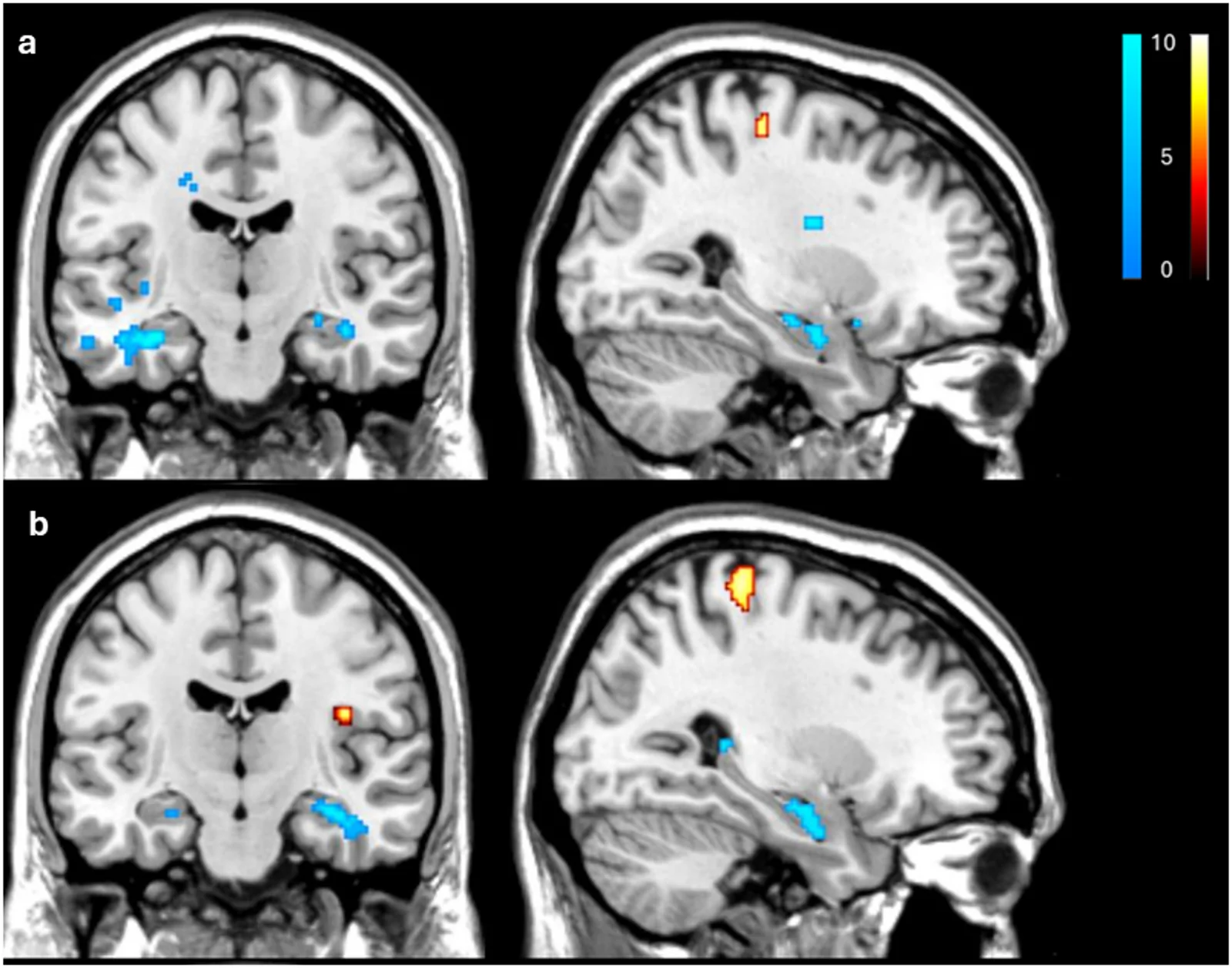
(**a**) Activation (orange) and deactivation (blue) during (self-object) touch in HC (T-test, p(FWE-corrected at the whole brain level) < 0.05) MNI [28 − 24 − 12]. (**b**) Activation (orange) and deactivation (blue) during (self-object) touch in AN (T-test, p(FWE-corrected at the whole brain level ) < 0.05). MNI [37 − 14 − 19]. Color bars display t-values. *Note* MNI coordinates are chosen to provide the clearest image of all relevant clusters.

*HC*: In line with previous findings, healthy participants showed the expected attenuation of self-produced touch. During (self-object) touch, HC displayed significant activation in the right primary somatosensory cortex (S1), and widespread deactivations in the superior temporal sulcus (STS), superior temporal gyrus (STG), bilateral insula, hippocampus, and amygdala (p(FWE) < 0.05). (Fig. 1a). For values, see Table 2.

*AN*: In contrast, women with AN showed increased activation in the right posterior insula and right S1 during (self-object) touch. Deactivations were observed in the amygdala, hippocampus, and STG bilaterally, as well as in the fusiform gyrus. In our region-of-interest (ROI), the right posterior insula, AN showed significant activation ([38 − 16 18], T = 6.56, *p* = 0.001). (Fig. 1b). For values, see Table 2.

*Other-touch condition*: For completeness, we also assessed other-touch relative to baseline. In HC, other-touch elicited robust activations across sensory and interoceptive regions (insula, thalamus, S1, fusiform gyrus, pACC), whereas AN showed more limited activations, mainly in medial frontal and hippocampal areas. Both groups also displayed widespread deactivations during other-touch, in frontal, insular, and parietal regions. (for values, see supplementary Table 1).

**Table 2:** Whole Brain Level Analysis for (self-object) touch.

Group	Type	Region	Hemisphere	Coordinates (MNI)	Cluster size	t-Value, *p*-value
AN	Activation	S1	Right	32 − 30 64	253	7.08, 0.000
		Insula	Right	38 − 16 18	23	6.56, 0.001
	Deactivation	Amygdala	Left	− 24 − 10 − 18	177	6.89, 0.000
		Hippocampus	Right	34 − 12 − 18	264	6.89, 0.000
		Amygdala	Right	24 − 8 − 18		6.80, 0.001
		Hippocampus	Left	− 36 − 26 − 18	155	6.76, 0.000
		Fusiform Gyrus	Right	44 18 − 32	85	6.86, 0.001
		STG	Left	− 50 − 2 − 16	16	4.93, 0.003
		STG	Right	50 8 − 29	10	6.04, 0.012
HC	Activation	S1	Right	26 − 24 56	13	6.18, 0.003
	Deactivation	Hippocampus	Right	36 − 26 − 8	140	7.67, 0.001
		Amygdala	Right	24 − 6 − 18	63	7.17, 0.003
		STG	Right	52 2 − 10	18	6.53, 0.013
		M1	Right	60 14 34	9	6.45, 0.015
		STS	Left	− 58 − 16 − 20	7	5.08, 0.015
		Insula	Right	32 10 − 12	17	6.33, 0.020
		STG	Left	− 46 − 16 − 8	3	6.17, 0.029
		Insula	Left	− 36 10 − 8	1	5.98, 0.045

*S1* primary somatosensory cortex, *STG* superior temporal gyrus, *STS* superior temporal sulcus, *M1* primary motor cortex.

P-values of FWE-correction for the whole brain at voxel-level.

### Differences between AN and HC

At the whole-brain level, no significant group differences emerged for (self-object) touch. However, in an exploratory analysis of extracted mean posterior insula ROI beta-values, AN showed greater mean activation than HC (AN: M = 0.01, SD = 0.34; HC: M = − 0.145, SD = 0.32; T = − 2.156, *p* = 0.034).

### Relationships between brain activation and body image disturbance

Posterior insula activation during self-touch was significantly associated with measures of body image disturbance.

**Fig. 2:**
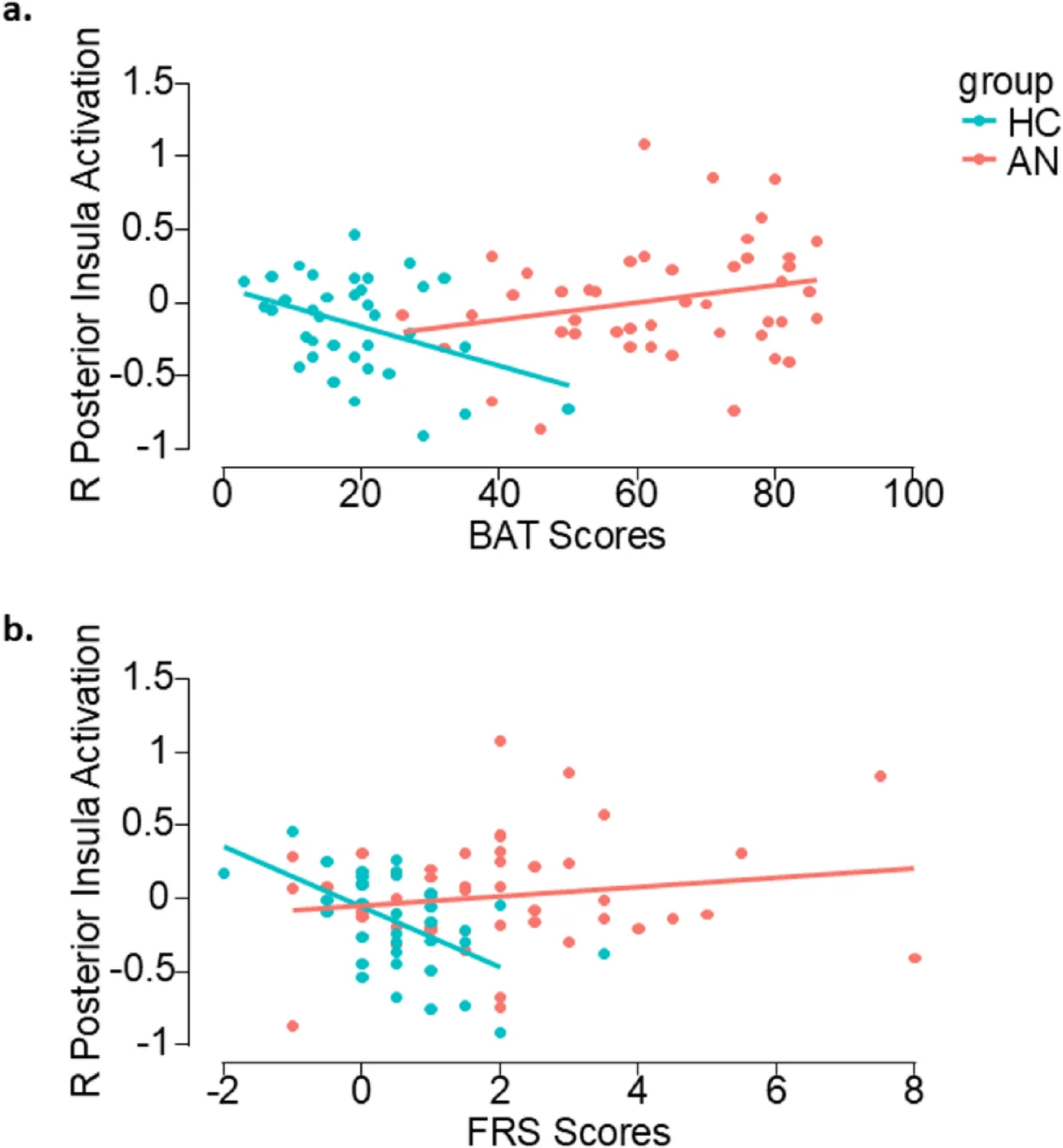
Scores from BAT (**a**) and FRS (**b**) correlated with the extracted mean beta values from posterior insula ROI during (self-object) touch, in opposite directions for the two groups. Insular activation (Y-axis) refers to the extracted ROI values.

A correlation was found between insular activation during self-touch (i.e. [self-object]) and BAT scores for both groups, but in different directions (Fig. 2a): there was a positive relationship in AN, (*r* = 0.369, *p* = 0.013), and a negative relationship in HC (*r *= − 0.389, *p* = 0.019). A similar pattern was found for the FRS (Fig. 2b): AN (*r* = 0.34, *p* = 0.022), and HC (*r *= − 0.506, *p* = 0.002).

The correlation coefficients differed significantly between groups for both FRS (Z = 4.016, *p *< 0.001) and BAT (Z = 3.211, *p *= 0.001).

No correlations were found between insular activation and EDE-Q scores (AN: *r* = 0.229, *p* = 0.131, HC: *r* = 0.098, *p* = 0.599) or any EDE-Q subscores (restraint, eating concern, weight concern, or shape concern).

For the other-touch condition ((other-object) contrast, supplementary Table 1), no correlations were found between insular activation and BAT (HC *r* = 0.02, *p* = 0.91; AN *r *= − 0.081, *p* = 0.596), FRS (HC *r* = 0.223, *p* = 0.197; AN *r*=-0.120, *p* = 0.434), or EDE-Q (HC *r* = 0.166, *p* = 0.364; AN *r *= − 0.156, *p* = 0.31).

## Discussion

Posterior insular responses to self-produced affective touch were associated with body image disturbance in opposite directions in individuals with AN and healthy controls, pointing to a qualitative difference in how self-touch is processed in relation to bodily self-perception across these groups.

In the AN group, we found right posterior insular activation during self-touch compared to object-touch. The control group did not show this insula activation during self-touch. An exploratory comparison of extracted ROI values confirmed greater mean activation in AN than HC, though this difference did not survive whole-brain FWE correction, indicating overlap in activation distributions between groups and warranting cautious interpretation. The key finding of the present study is therefore not a simple group difference in insular activation, but rather the divergent relationship between that activation and body image disturbance across groups.

Insular activity during self-touch in AN could reflect a prediction error during a self-produced tactile event, a percept which in healthy populations is usually highly predictable and attenuated (typically resulting in bilateral insular deactivation)^25,36^. Prediction errors during heat and taste perception tasks have been shown to be heightened in the right insula in AN^37,38^.

Two accounts may explain this heightened response. First, it may reflect a prediction error: if the bodily self-model is distorted, self-produced touch would generate a mismatch between expected and actual somatosensory input, increasing insular activity. Second, it may reflect a compensatory mechanism by which the brain attempts to reconcile conflicting multisensory signals about body size. These accounts are not mutually exclusive. A prediction error is a signal that could trigger compensatory processing, but they carry different implications. If prediction error is primary, insular hyperactivation should diminish as body image normalizes with treatment. If compensatory mechanisms dominate, longitudinal studies might instead reveal that insular hyperactivation precedes symptomatic improvement. Cross-sectional fMRI data, as used here, cannot differentiate between these possibilities, and future longitudinal or intervention designs are needed.

Heightened insular activity during self-touch could alternatively reflect an emotional response (of anxiety or disgust, for example) to touching one’s own body, as has been suggested in AN^39,40^ and bulimia^41^. The posterior insula has been implicated in emotion regulation in addition to the functions listed above; however, in this case we would also expect to see increased activation in other emotion processing areas. Instead, we find bilateral amygdala deactivations in both groups at the whole brain level during (self-object) touch, rendering the implication of insular activation as a result solely from an emotional response unlikely. Amygdala deactivation during self-produced touch has been found previously in healthy populations^25^, and indicates a decrease in emotional salience and valence during self-produced touch.

In AN, more activation corresponded to greater BID severity across both questionnaire measures, i.e. greater activation corresponded to greater body dissatisfaction (BAT) and larger perceived-actual body size discrepancy (FRS). This is in line with a previous study on the visual modality which found increased insular activation in AN when comparing images of self and other, and correlated with body satisfaction^17^. Structural alterations in the posterior insula are also associated with body image disturbances, and increased gray matter in the posterior insula in AN is associated with longer duration of the disorder and body shape dissatisfaction^22^. Some studies find decreased resting state connectivity in the posterior insula in AN^42^, potentially reflecting a pathophysiological calibration^43^ and/or integration^19^ of internal (interoceptive, homeostatic) and external (somatic, visuospatial) bodily signals, while another study found increased connectivity between the posterior insula and the default mode network in AN, potentially indicating altered modification of somatosensory information^44^.

Controls displayed the opposite relationship: increased activation was associated with decreased body image disturbances; i.e., more activation corresponded to increased body satisfaction. This may reflect more efficient integration of self-related somatosensory signals in individuals with an accurate bodily self-model.

We found that insular activation did not correlate with EDE-Q scores in either group, which does not align with previous findings of increased right posterior insular gray matter correlating with EDE-Q scores in AN^21^. The EDE-Q measures eating disorder symptomatology rather than body dissatisfaction, suggesting that the activation in response to our self-touch task is not related to the physical symptoms of AN but rather body dysmorphia and the mental representation of the body.

We found more activations in the right posterior insula during the (other-object) touch condition in HC than in AN. We did not find correlations between this activation and BAT or FRS scores for either group, implicating this region in processing of self-generated touch, specifically, and not in the processing of affective skin-to-skin touch in general.

A limitation with these findings, and with AN research in general, is that it is difficult to disentangle the effects of malnourishment from underlying differences in neural mechanisms. Importantly, the average BMI of our participants was within the lower healthy range, however one cannot discount the symptomatology of AN and the effects that a starvation state may have on brain function and behavior. Additionally, the cross-sectional design prevents causal inferences about the relationship between insular function and BID.

In summary, individuals with AN showed increased right posterior insular activation during self-produced affective touch, and this activation was positively associated with BID severity, opposite to the pattern in healthy controls. The group difference in activation itself was modest, but the divergent correlations with body image measures point to a difference in how self-touch is processed in relation to bodily self-perception in AN. This difference may reflect heightened prediction errors during self-produced touch arising from a distorted bodily self-model, though longitudinal studies are needed to evaluate this interpretation. Future therapeutic approaches incorporating self-touch or other self-produced sensory input may hold promise for addressing the embodiment disruptions central to AN.

## Methods

### Participants

The study was approved by the Regional Ethical Board of Linköping (2017/443 − 31, 2018/444 − 32) and the Swedish Ethical Review Authority (2019–02821), and conducted in accordance with the Declaration of Helsinki. Participants provided written informed consent .

Adults (aged 18–35) with AN were recruited through the Child and Adolescent Psychiatric Clinic (18–24 years) and the Psychiatric clinic (25–35 years) in Linköping, Region Östergötland. Clinical staff first informed potential participants of the study. Interested participants were then contacted by a research nurse (not involved in patients’ treatment) who gave further verbal and written information about the study and screened the participants for the inclusion and exclusion criteria. Participants were included on the conditions: DSM-5 diagnosis of anorexia nervosa, including atypical anorexia nervosa and unspecified (all restrictive type), BMI ≤ 20 kg/m2, and MRI-compatibility. Participants were either free of psychotropic medications or on stable (at least three months on the same dose) medication with antidepressants. On-demand use of mild anxiolytics and hypnotics, treatment with central stimulants (if avoided on MRI day) were also accepted. Exclusion criteria were: psychotic disorder, bipolar disorder, substance use disorder, ongoing treatment with antipsychotics or tricyclic antidepressants, previous severe head injury, birth before 33 weeks’ gestation, hearing impairment, previous epilepsy or seizure, claustrophobia, pregnancy, and cognitive disabilities. In total 44 participants were included in the current study.

Healthy controls (HC) were recruited online through social media advertisements and on the university website as well as through our local participant database. Participants were included based on Swedish fluency, acceptance of skin-to-skin touch on the arm, and willingness to undergo an MRI scan. Exclusion criteria were assessed via a standardized phone interview screening^45^ and included any psychiatric diagnoses or medication, history of disordered eating, excessive substance use, chronic pain, and any health issues related to touch perception or MRI safety. In total 40 participants were recruited and, with one participant not completing all of the questionnaires, 39 participants were included in the analyses for HC.

### Questionnaires

Participants completed the Body Attitude Test (BAT;^34^, Eating Disorder Examination Questionnaire (EDE-Q^35^, and Figure Rating Scale (FRS;^46^, to assess body image disturbances and the severity of eating disorder symptomatology.

The FRS score used in analyses reflects the discrepancy between the figure selected as representing the participant’s current body size and the figure selected as the ideal/actual body size, such that higher scores indicate greater perceived-actual body size discrepancy.

### Touch task

Participants performed the self-other-touch task as standardized in previous literature^25,47^: they lay in an fMRI with their left arm across their abdomen and a pillow on the abdomen beside the arm. The right arm was propped up as close to the left arm and pillow as possible using pillows. The touch task consisted of three conditions: self-touch, other-touch, and object-touch. During self-touch, participants gently stroked their left forearm with their right hand. During other-touch, participants were gently stroked on the left forearm by a female researcher. During object-touch, participants stroked a pillow with their right hand. The object-touch condition was used as a control for the movement in the self-touch condition. Goggles displayed instructions for the task. Instructions read (in Swedish), ‘Active, please stroke your arm’, ‘Passive, the researcher will stroke your arm’, ‘Active, please stroke the object’. Instructions were provided on a white background for three seconds before turning green to indicate when the condition should begin. Each condition lasted for 12 s, with 12 s of rest in between. Conditions were repeated ten times each, for a total of 30 trials in random order.

### fMRI

A 3.0 Tesla scanner (Prisma; Siemens) with a 64-channel head coil was used to acquire T1-weighted anatomical images (repetition time = 2300 ms; echo time = 2.36 ms; flip angle = 8°; field of view = 288 × 288 mm²; voxel resolution = 0.87 × 0.87 × 0.90 mm³) and T2\*-weighted echo-planar images (EPIs) containing 48 multiband slices (repetition time: 1030 ms; echo time: 30 ms; slice thickness: 3 mm; matrix size: 64 × 64; field of view: 192 × 192 mm²; in-plane voxel resolution: 3 × 3 mm²; flip angle: 63°). Statistical parametric mapping (SPM12; Wellcome Department of Imaging Neuroscience) in Matlab R2018b (MathWorks) was used to analyze fMRI data. Preprocessing steps included: Correction for motion using SPM’s realign-module registering to the mean EPI after a first realignment (quality = 0.9, separation = 4, smoothing = 5 full width at half-maximum kernel, interpolation with 4th degree B-Spline), coregistration of the anatomical image and mean EPI using normalized mutual information, segmentation of the T1 image using the unified segmentation approach^48^, and spatial normalization of T1 and EPIs to the Montreal Neurological Institute T1 template (using forward deformations from the segmentation step, voxel size 2 × 2 × 2 mm³ for resampling, and 4th Degree B-Spline for interpolation). All functional images were spatially smoothed with an isotropic Gaussian kernel of 6-mm full width at half-maximum.

### Analysis

At the single-subject level, regressors of interest consisted of self-touch, other-touch, and object-touch blocks. Regressors of no-interest were added for the cue phase (separately for each block), and arm movements after each active block (1 s) when subjects moved the arm back into the resting position. To account for movement-associated variance, realignment parameters and their first temporal derivates were included as regressors of no interest, as well as a regressor censoring scans with more than 1 mm scan-to-scan movement. For the analysis here, our hypothesis concerned the self-touch condition because object-touch controls for the active movement component. Therefore, contrast maps were generated for the self-touch vs. object-touch condition at the single subject level. The interpersonal dimension of other-touch, which is also relevant but distinct, is reported separately for completeness.

At the group-level, one sample t-test for the self-object contrast was calculated for evaluation of the AN group and extraction of beta-parameters. For completeness, a one-sample t-test per group was evaluated for the other-touch condition as well. Two sample t-tests were used for between-group comparisons. For whole-brain analyses, results were corrected for multiple comparisons using the family-wise error (FWE) correction at the voxel-level^49^. An anatomical region of interest (ROI) mask for the right (unilateral) posterior insula was derived from^50^ and used for extraction of mean beta parameters for exploratory group comparisons and correlational analyses with values from EDE-Q, BAT, and FRS questionnaires.

Due to inequality of variance, Mann-Whitney tests were performed for between-group measures for the questionnaires. Fisher’s r-to-z transformation was used to test differences in correlation coefficients between groups.

## Supplementary Information

Below is the link to the electronic supplementary material.

Supplementary Material 1

## Data Availability

The datasets analyzed during the current study are not publicly available due to ethical considerations, but are available from the corresponding author on reasonable request.
