# Supplementary material for: Attenuation of self-produced touch in the posterior insula reflects body image disturbances in anorexia nervosa
Source: Sci Rep. 2026 Sep 26;16:29913. doi: 10.1038/s41598-026-71154-5 (PMC13637035; doi:10.1038/s41598-026-71154-5)
Supplement: Supplementary file 1 [file 41598_2026_71154_MOESM1_ESM.docx]

**Supplement**

| Group | Type | Region | Hemisphere | Coordinates (MNI) | T-value / p-value |
| --- | --- | --- | --- | --- | --- |
| AN | Activation | PreSMA | Right | [8 6 44] | T=13.16, p=0.000 |
|  |  | MFG | Right | [32 38 28] | T=6.69, p=0.000 |
|  |  | MFG | Left | [-28 38 30] | T=6.28, p=0.001 |
|  |  | Amygdala | Left | [-32 2 -28] | T=5.76, p=0.004 |
|  |  | Hippocampus | Left | [-34 28 -20] | T=5.28, p=0.003 |
|  |  | Hippocampus | Right | [34 -12 -18] | T=6.89, p=0.024 |
|  | Deactivation | Frontal pole | Left | [-4 68 20] | T=9.17, p=0.000 |
|  |  | IFG | Left | [-56 30 16] | T=8.39, p=0.000 |
|  |  | IPL | Left | [-48 68 48] | T=8.18, p=0.000 |
|  |  | IPL | Right | [54 -60 48] | T=6.00, p=0.002 |
|  |  | OFC | Left | [-26 26 -16] | T=5.54, p=0.009 |
|  |  | OFC | Right | [28 30 -18] | T=5.27, p=0.023 |
| HC | Activation | Insula | Right | [36 -16 20] | T=15.29, p=0.000 |
|  |  | ACC | Right | [4 34 6] | T=11.56, p=0.000 |
|  |  | SFG | Left | [-8 58 20] | T=11.15, p=0.000 |
|  |  | IPL | Left | [-50 -74 32] | T=10.26  p=0.000 |
|  |  | Insula | Left | [-30 12 -14] | T=9.08, p=0.000 |
|  |  | SPL | Right | [20 -38 68] | T=8.12, p=0.000 |
|  |  | Amygdala | Left | [-26 -10 -16] | T=7.77, p=0.000 |
|  |  | Hippocampus | Left | [-30 -22 -14] | T=7.15, p=0.000 |
|  |  | Thalamus | Right | [18 -24 4] | T=7.37, p=0.000 |
|  |  | M1 | Left | [-64 -10 38] | T=6.12,  p=0.002 |
|  |  | S1 | Right | [38 6 50] | T=5.81,  p=0.006 |
|  |  | S1 | Left | [-50 0 54] | T=5.70,  p=0.008 |
|  |  | Fusiform gyrus | Right | [42 -48 -16] | T=5.69,  p=0.009 |
|  |  | MFG | Left | [-34 32 52] | T=5.22,  p=0.043 |
|  | Deactivation | M1 | Left | [-30 -24 62] | T=16.85,  p=0.000 |
|  |  | Thalamus | Left | [-14 -22 4] | T=15.20,  p=0.000 |
|  |  | Insula | Left | [-44 -22 20] | T=7.53,  p=0.000 |
|  |  | IPL | Right | [42 -32 48] | T=6.48, p=0.000 |
|  |  | IFG | Left | [-56 4 30] | T=5.41, p=0.023 |
|  |  | Substantia nigra | Left | [-8 -20 -12] | T=5.37, p=0.026 |

*Table 1: Whole-Brain analyses of Other-touch. SMA, supplementary motor area; MFG, medial frontal gyrus; IFG, inferior frontal gyrus; IPL, inferior parietal lobe; OFC, orbitofrontal cortex; ACC, anterior cingulate cortex; SFG, superior frontal gyrus; SPL, superior parietal lobe; M1, primary motor cortex; S1, primary somatosensory cortex.*

| **Subscale** | **Mean** | | **SD** | | **t** | **df** | **p** |
| --- | --- | --- | --- | --- | --- | --- | --- |
|  | **HC** | **AN** | **HC** | **AN** |  |  |  |
| Restraint | 0.40 | 3.49 | 0.82 | 1.59 | −10.07 | 76 | < .001 |
| Eating concern | 0.10 | 3.11 | 0.22 | 4.10 | −4.13 | 76 | < .001 |
| Shape concern | 0.20 | 2.29 | 0.27 | 1.59 | −7.34 | 76 | < .001 |
| Weight concern | 0.09 | 1.87 | 0.24 | 1.95 | −5.13 | 76 | < .001 |
| EDE-Q total | 1.01 | 10.76 | 1.55 | 6.61 | −8.17 | 76 | < .001 |

*Table 2: EDE-Q subscale scores. HC = healthy controls; AN = anorexia nervosa; SD = standard deviation. All p-values are two-tailed independent samples t-tests.*
